# Molecular mechanisms of ferroptosis and its role in cancer therapy

**DOI:** 10.1111/jcmm.14511

**Published:** 2019-06-24

**Authors:** Tao Xu, Wei Ding, Xiaoyu Ji, Xiang Ao, Ying Liu, Wanpeng Yu, Jianxun Wang

**Affiliations:** ^1^ School of Basic Medical Sciences Qingdao University Qingdao China; ^2^ Center for Regenerative Medicine, Institute for Translational Medicine, College of Medicine Qingdao University Qingdao China; ^3^ Department of Comprehensive Internal Medicine, Affiliated Hospital Qingdao University Qingdao China

**Keywords:** cancer therapy, drug resistance, ferroptosis, programmed cell death, small molecules

## Abstract

Ferroptosis is a newly defined programmed cell death process with the hallmark of the accumulation of iron‐dependent lipid peroxides. The term was first coined in 2012 by the Stockwell Lab, who described a unique type of cell death induced by the small molecules erastin or RSL3. Ferroptosis is distinct from other already established programmed cell death and has unique morphological and bioenergetic features. The physiological role of ferroptosis during development has not been well characterized. However, ferroptosis shows great potentials during the cancer therapy. Great progress has been made in exploring the mechanisms of ferroptosis. In this review, we focus on the molecular mechanisms of ferroptosis, the small molecules functioning in ferroptosis initiation and ferroptosis sensitivity in different cancers. We are also concerned with the new arising questions in this particular research area that remains unanswered.

## 1 INTRODUCTION

Ferroptosis is a newly coined non‐apoptotic programmed cell death process that was discovered via a chemical screen.^1^ The general initiation mechanisms of ferroptosis have partially been elucidated with the research going further. The metabolism of cysteine, polyunsaturated fatty acids (PUFAs) and iron are all closely correlated with ferroptosis initiation. Multiple signalling pathways as well as the cell organelles have also been found to involve in the ferroptosis regulation. Moreover, a series of small molecules have been found to be able to induce ferroptosis in a wide range of cancer cells. These findings provide the possibility of cancer therapies through genetic or pharmacological interference with ferroptotic cell death, which is of great interest in both scientific research and medicine. Different kinds of cancers seem to have various sensitivities to ferroptosis. A clear understanding of ferroptosis sensitivity in cancers from different tissues will also benefit the clinical practice in applying ferroptosis to cancer therapy. In this review, we summarize the general initiation mechanisms of ferroptosis, the small molecules involved in ferroptosis initiation and the signalling pathways as well as the cell organelles involved in ferroptosis regulation. Moreover, we talk about the potential application of ferroptosis in overcoming cancer cell drug resistance from several aspects.

## 2 MAIN TEXT

### 2.1 Ferroptosis represents a new way of cell death process

Ferroptosis is different from other programmed cell death from several aspects. Lipid peroxides accumulation and iron dependency are the two major features of ferroptosis. Ferroptotic cell death also has unique morphological and bioenergetic features including shrunken mitochondria, increased mitochondrial membrane density, disruption of membrane integrity and depletion of intracellular NADH, but not ATP levels. The induction of ferroptosis depends on ATP production but does not require caspase activation. Moreover, ferroptosis is not sensitive to the inhibition of RIP1/RIP3 or Cyclophilin D, which are key regulators of necrosis, and inhibition of autophagy by 3‐MA does not modulate this cell death process.^1^ The evidence suggests that ferroptosis is a completely new way of cell death.

### 2.2 The mechanisms of ferroptosis initiation

#### 2.2.1 Cysteine metabolism plays a central role in ferroptosis initiation

**Figure 1:**
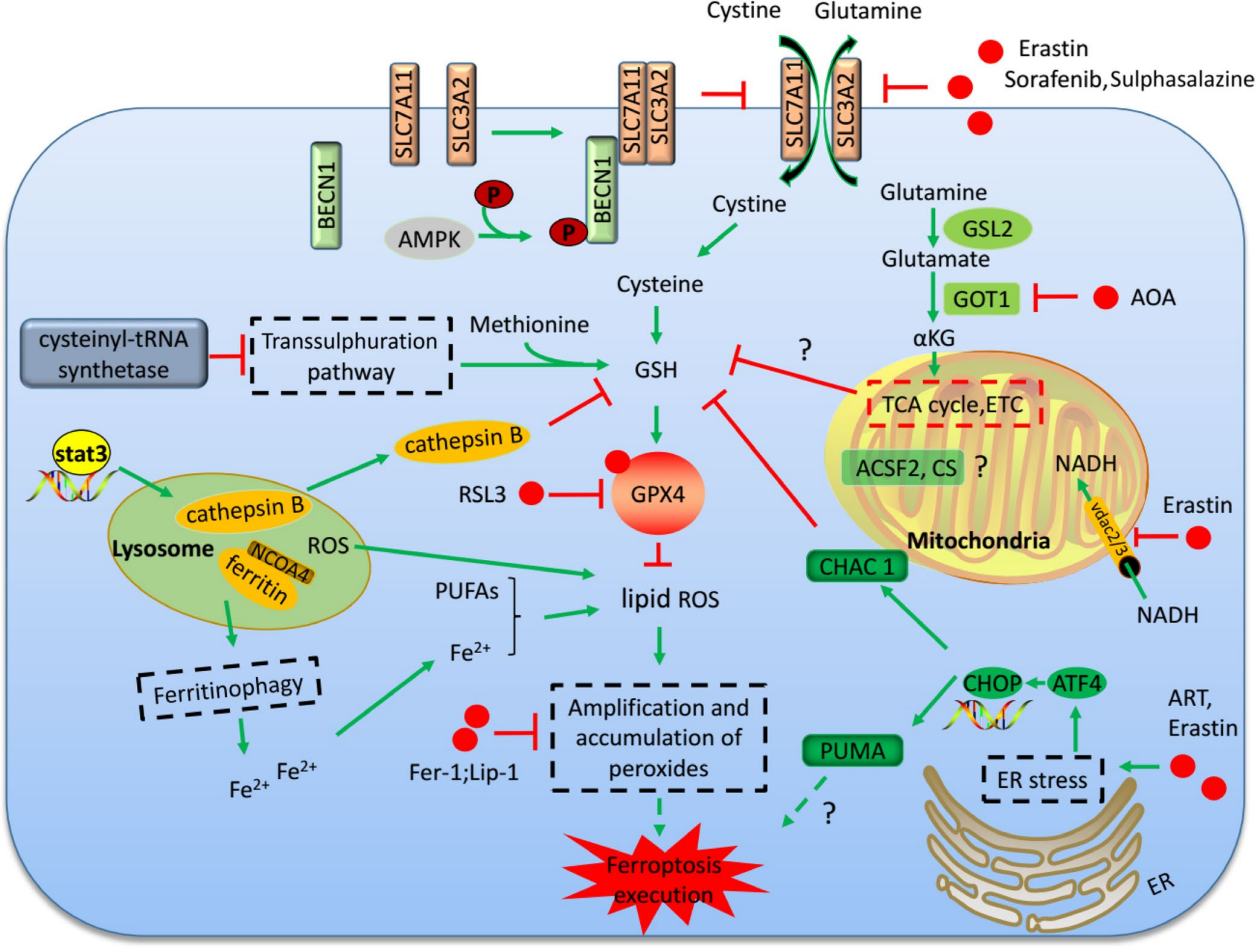
Mechanisms of ferroptosis induction. Inhibition of system *x*_c_^−^ deprives cellular cysteine, leading to GSH deletion and GPX4 inactivation. GSH can be synthesized from methionine through the transsulphuration pathway which is inhibited by cysteinyl‐tRNA synthetase. RSL3 inhibits the activity of GPX4 by covalent binding with GPX4. GPX4 inactivation leads to the accumulation of lipid peroxides and final ferroptosis. Enzymes (GLS2 and GOT1) involved in glutaminolysis regulate ferroptosis process. The tricarboxylic acid (TCA) cycle promotes cellular GSH deletion and leads to ferroptosis in combination with cysteine deprivation. The mitochondrial genes (ACSF2, CS) are all involved in ferroptosis regulation. ER stress induced by ferroptotic reagents promotes ferroptosis through ATF4‐dependent CHAC 1 expression. Lysosome is also involved in ferroptosis induction through autophagy process or cathepsin B release. Lysosome ROS contributes to the lipid ROS production

Erastin is among the small molecules identified in chemical screen that induce ferroptosis in oncogenic RAS mutation cell lines. Exploration of the targets of erastin links cysteine metabolism to ferroptosis initiation. Glutamate‐cystine antiport system *x*_c_^−^ is the most important target of erastin during erastin‐induced ferroptosis (Figure 1).^1^ System *x*_c_^−^ transports cystine, the major form of cysteine in the atmosphere, into the cells by exchange of glutamate at a 1:1 ratio. The inhibition of system *x*_c_^−^ deprives the cellular cysteine, leaving it unavailable for GSH synthesis (Figure 1).^2^ GSH plays a major role in cellular antioxidant defences. Depletion of GSH leads to the accumulation of lipid ROS, protein or membrane damage and subsequent ferroptotic cell death.^3^

β‐mercaptoethanol changes the cystine into cysteine which is transported into the cell, bypassing the system *x*_c_^−^ (Figure 1).^4^ β‐Mercaptoethanol treatment suppresses the cell death induced by system *x*_c_^−^ inhibition and cysteine deprivation.^5^ Some cell types are resistant to the erastin‐induced cell death possibly because these cells can obtain cysteine by alternative means. For example, knockdown of the cysteinyl‐tRNA synthetase activates the transsulphuration pathways through which the cells biosynthesize cysteine from methionine and resist to erastin‐induced ferroptosis (Figure 1).^6^

There were also some other targets of erastin identified in the affinity purification. SLC7A5 was also identified as an erastin binding protein by affinity assays. SLC7A5 can also bind SLC3A2 to form amino acid transporters (system L) of large, neutral amino acids. However, the inhibition of system L‐mediated amino acid uptake by erastin does not contribute directly to ferroptosis. The binding of erastin to the SLC7A5 likely interferes with cystine uptake by the SLC3A2/SLC7A11 complex in trans.^1^ Erastin can also target the mitochondrial‐resident voltage‐dependent anion channel‐2 (VDAC2). Knockdown of VDAC2 or VDAC3 by RNAi attenuates erastin‐induced ferroptosis. Erastin‐VDAC2 interaction inhibits the permeability of VDAC2 to endogenous substrates, such as NADH and decrease the NADH oxidation in cancer cells, which induces mitochondrial dysfunction and the release of oxidative species (Figure 1). However, both VDAC2 and VDAC3 are necessary, but not sufficient, for erastin‐induced cell death showed by the knockdown or overexpression studies.^7^

#### 2.2.2 GPX4 inactivation is causative for lipid peroxides accumulation

RSL3 is another molecule for ferroptosis initiation found in the chemical screen.^1^ The target for RSL3 was investigated by proteomic analysis of an affinity pull‐down assay, and GPX4 was found to be a direct target of RSL3.^8^ GPX4 can prevent the toxicity of lipid peroxides by its enzyme activity and maintain the homeostasis of membrane lipid bilayers (Figure 1).^9^ RSL3 inhibits the activity of GPX4 by covalent bonding with GPX4 and leads to lipid peroxides accumulation (Figure 1). Ferroptosis induced by RSL3 treatment is similar to that of GPX4 inactivation, further supporting that RSL3 induces ferroptosis through GPX4 inhibition. GSH is the co‐factor of GPX4 in catalysing peroxides into alcohols.^10^ GSH depletion caused by cysteine deprivation directly inactivates GPX4 and leads to subsequent induction of ferroptosis (Figure 1). Knockout of GPX4 in mice leads to embryonic lethality and mass lipid peroxides accumulation.^11^ GPX4‐deficient MEFs show resistance to cell death induced by erastin, suggesting this cell death is ROS dependent.^12^ Moreover, apoptosis does not occur in the GPX4 knockout mice, which further confirms ferroptosis as the leading cause of the lethality.^13^

#### 2.2.3 The selective lethality of ferroptotic compounds in different cancer cell lines

Ferroptosis was initially defined in RAS‐mutated cancer cells. Many different types of cancer cells with RAS mutation show sensitivity to ferroptosis induction. One explanation for the close relationship between RAS signalling and ferroptosis may be that activation of RAS can increase intracellular iron through the activation of transferrin receptor 1 (TFR1) and suppression of the iron storage proteins.^14^ However, the mutated RAS gene seems dispensable for ferroptosis initiation. Some cancer types without RAS gene mutation are also sensitive to ferroptosis induction. Furthermore, the rhabdomyosarcoma engineered to express the mutated RAS even confers resistance to erastin‐ or RSL3‐induced cell death.^15^ Leukaemia cells without RAS mutation also showed great sensitivity to ferroptosis induction.^1^

Further studies showed the different ferroptosis sensitivities in cancer cells from different tissues through a large‐scale profiling experiment that screened four ferroptotic reagents (erastin, RSL3, ML210 and ML162) against a panel of 860 cancer cells. Researchers concluded that cancer cell lines derived from haematopoietic and lymphoid, CNS, autonomic ganglia, ovary, soft tissue, kidney and bone tissue were the most sensitive to the four ferroptotic reagents. Cell lines derived from the oesophagus, upper respiratory tract, stomach, pancreas, breast, skin and large intestine were generally insensitive to the four ferroptotic reagents. Cancer cell lines of non‐epithelial origin were more sensitive to ferroptotic reagents than cancer cell lines of epithelial origin. Cancer cells in a high‐mesenchymal state were more likely to undergo ferroptosis.^16^

#### 2.2.4 Glutaminolysis is indispensable for cysteine deprivation‐induced ferroptosis initiation

Recent studies have shown that glutaminolysis played an indispensable role in ferroptosis initiation in mouse embryonic fibroblasts (MEFs).^17^ Glutamine is degraded via glutaminolysis and the tricarboxylic acid (TCA) cycle.^17^, ^18^ Further evidence demonstrates that glutaminolysis metabolite αKG or its downstream metabolites during the TCA cycle are required for the induction of ferroptosis (Figure 1).^17^ Several enzymes involved in glutaminolysis have been revealed to play important roles in ferroptosis initiation. Transaminase converts glutamate into αKG through the transamination process.^19^ The inhibitor of transaminases, amino‐oxyacetate (AOA), was found to inhibit ferroptosis in MEFs. Knockdown of the transaminase GOT1 could also inhibit cysteine deprivation‐induced ferroptosis in MEFs (Figure 1).^17^, ^20^ However, the role of glutaminolysis in ferroptosis regulation is more complex. Glutamate dehydrogenase 1 (GLUD1) converts glutamate into αKG through the glutamate deamination. However, RNAi‐mediated knockdown of GLUD1 failed to inhibit ferroptosis initiation.^17^ Both Glutaminases 1 (GLS1) and Glutaminases 2 (GLS2) catalyse the conversion of glutamine into glutamate. Studies demonstrated that only GLS2 is involved in the regulation of ferroptosis (Figure 1). Further study illustrated that GLS2 is the transcriptional target of p53 and is up‐regulated during p53‐dependent ferroptosis.^20^, ^21^ Moreover, only in combination with glutamine can cysteine deprivation induce ROS accumulation, lipid peroxidation and ferroptosis. This phenomenon was explained by recent study from Gao et al,^22^ whose work discovered a role for mitochondria in cysteine deprivation‐induced ferroptosis in both MEFs and HT1080 cells. Under cysteine deprivation, glutaminolysis will promote mitochondrial respiration and the rapid exhaustion of GSH by GPX4, inducing potent ferroptosis. However, when glutaminolysis is inhibited, GSH turnover rate is slowed and ferroptosis is not induced even when cysteine is deprived. (Figure 1).^22^

Elevated glutaminolysis has been observed in most cancer cells to satisfy their bioenergetic requirements. However, high rate of glutaminolysis showed vulnerability of cancer cells for its role in promoting ferroptosis induction in cancer cells. Glutaminolysis in combination with cysteine deprivation induces potent ferroptotic cell death and will revolutionize the anti‐tumour strategy.

#### 2.2.5 PUFAs and cellular iron are essential for lipid peroxide accumulation

**Figure 2:**
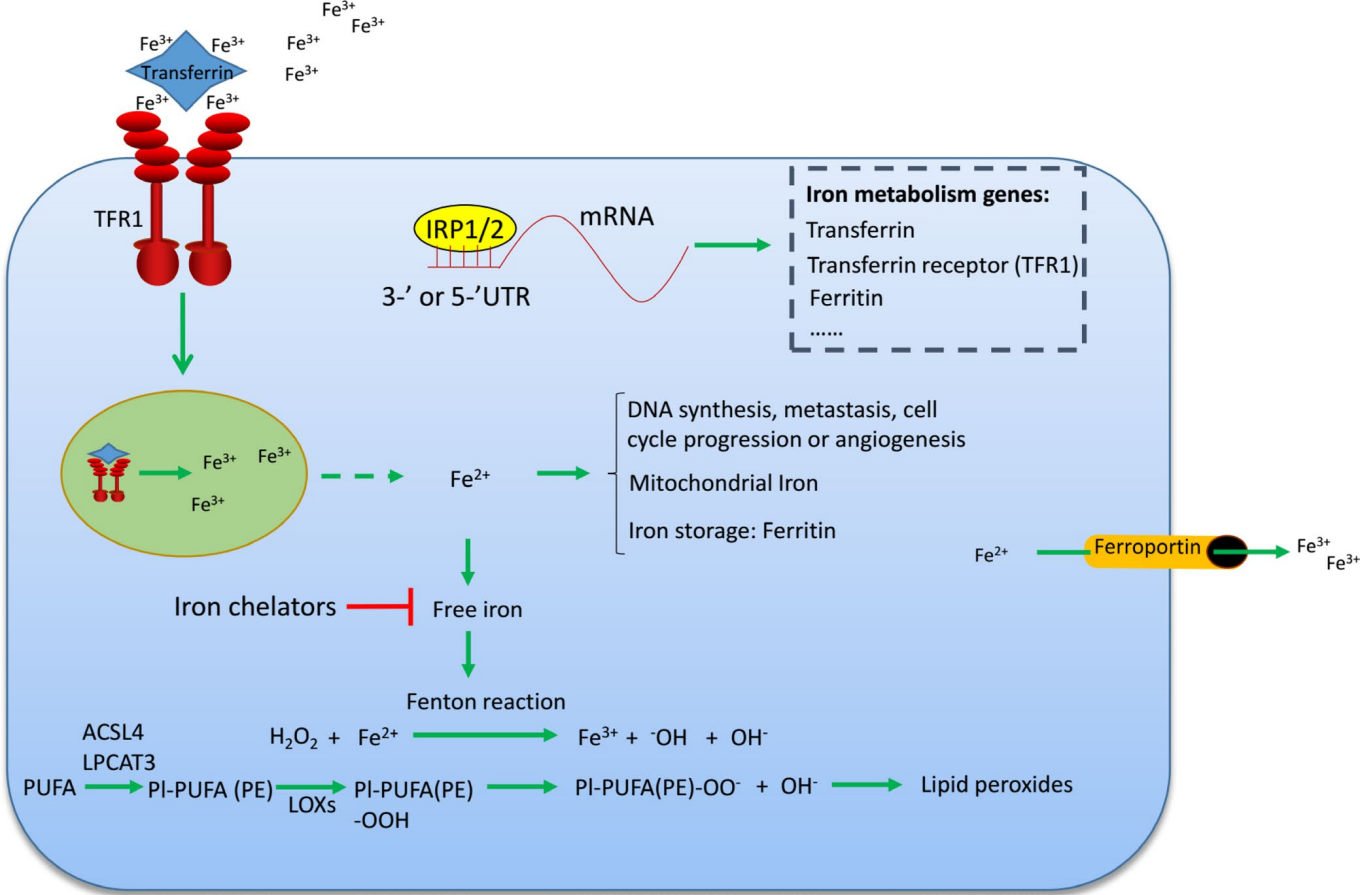
Iron metabolism and lipid peroxides accumulation. Transferrin transports the iron into cells by the TFR1‐mediated endocytosis. Ferroportin exports and decreases the cellular iron. Cellular iron is involved in the normal life process such as DNA synthesis, metastasis, cell cycle progression, angiogenesis or mitochondrial iron metabolism. Ferritin is the iron storage protein in the cells. Only the free iron is involved in the Fenton reaction. Fe^2+^ promotes the lipid peroxides accumulation through Fenton reaction and lipid oxidation. IRP1/2 regulates the iron metabolism genes by binding with the 3‐′ or 5‐′ UTR of the mRNAs

Polyunsaturated fatty acids increase membrane fluidity and are important for the adaption of original life to the environment. However, PUFAs can be oxidized by intracellular ROS and produce the lipid peroxides that promote the induction of ferroptosis (Figures 1 and 2).^23^ The activity of lipoxygenases (LOXs) could catalyse PUFA‐containing phospholipids into pro‐ferroptotic lipid peroxidation.^24^ A CRISPR‐based genetic screen identified two lipid metabolism regulators, lysophosphatidylcholine acyltransferase 3 (LPCAT3) and acyl‐CoA synthetase long‐chain family member 4 (ACSL4) that promote GPX4 inhibition‐induced ferroptosis in KBM7 cells.^25^ The catalysed functions of ACSL4 and LPCAT3 are responsible for membrane phospholipid insertion and polyunsaturated fatty acid remodelling (Figure 2).^26^, ^27^ Knockout of ACSL4 resulted in marked resistance to ferroptosis induced by GPX4 deficiency.^28^

Iron serves as the essential component of many enzymes involved in DNA synthesis, metastasis, cell circle progression or angiogenesis (Figure 2). However, iron is also a redox‐active reagent and promotes ROS production via Fenton reaction (Figure 2).^29^ A free iron requirement is the fundamental property of ferroptosis and almost all lipid peroxides could be diminished by iron chelators, linking the iron metabolism process closely with the ferroptosis initiation.^30^ A series of genes are involved in the iron metabolism. IRP1 and IRP2 are the sensors of intracellular iron and govern iron transport, storage and turnover through controlling the expression of a series of iron metabolism genes. IRP‐1 and IRP‐2 control the expression of its target genes by binding to a stem‐loop structure located in the 3′‐ or 5′‐untranslated region of the mRNA according to the intracellular iron level. The binding of IRP‐1 and IRP‐2 to mRNA stabilizes it and increases its expression or changes its localization (Figure 2).^31^ Transferrin is the transporter of iron, and it imports iron into the cell from the extracellular environment through recognition by TFR1 (Figure 2). Both transferrin and TFR1 are the targets of IRP1 and IRP2 and are required for ferroptosis induction.^32^ Ferritin is also the target of IRP1 and IRP2. Ferritin binds to the free iron and makes it unavailable and therefore functions to prevent ferroptosis.^33^, ^34^ Ferroportin is responsible for iron export from the cells and is the negative regulator of ferroptosis (Figure 2).^35^ Iron chelators have long been applied to the development of anti‐tumour strategies.^36^ However, the iron requirement in ferroptosis induction redefined the role of iron chelators in cancer treatment. Future studies are still needed to validate the iron chelation strategies in cancer therapy under different conditions.

### 2.3 The emerging roles of different organelles involved in ferroptosis regulation

The organelles are the important components of the cell and function to maintain intracellular homeostasis. However, dysfunction of the cell organelles under stress conditions will promote the cell death process. The mitochondria, lysosome and the endoplasmic reticulum (ER) have all been demonstrated to play important roles in ferroptosis regulation. Here, we talk about the specific role of different organelles in ferroptosis regulation.

#### 2.3.1 The role of mitochondria in ferroptosis

Mitochondria is the energy provider of the cell and has long been considered to be closely related to the programmed cell death process.^37^ Nonetheless, the role of mitochondria in ferroptosis remains highly controversial. Dixon's work showed that ferroptosis could occur in cells lacking a functional mitochondrial electron transport chain (ETC) in HT‐1080 cells. However, Gao et al showed that depletion of the mitochondria through parkin‐mediated mitophagy dramatically decreased the sensitivity of cells to cysteine deprivation‐induced ferroptosis as mentioned above. Inhibition of mitochondrial TCA cycle or electron transport chain attenuates cysteine deprivation‐induced ferroptosis (Figure 1). Despite glutaminolysis and the TCA cycle in the mitochondria, mitochondrial lipids seem also to be important sources for lipid peroxides production during ferroptosis. However, they showed that mitochondria only played a role in cysteine deprivation‐induced ferroptosis, but not GPX4 inhibition‐induced ferroptosis. This is possibly because the mitochondria function upstream of GPX4 to promote the exhaustion of GSH under cysteine deprivation conditions (Figure 1).^22^ The important roles of mitochondria in ferroptosis regulation were also confirmed in cardiomyocytes. In the doxorubicin‐induced myocardial ferroptosis, lipid peroxidation and non‐haem iron were specifically increased in the mitochondria, but not in the cytoplasm.^38^ What's more, mitochondrial fatty acid metabolism genes including citrate synthase (CS) and acyl‐CoA synthetase family member 2 (ACSF2) are possibly required for erastin‐induced ferroptosis. (Figure 1).^1^ All these findings link the mitochondria closely with ferroptosis induction. Opposite results on the roles of mitochondria in ferroptosis may be due to the different methods for cell death measurement. The cell respiration assays may not be suitable for the study of the role of mitochondria in ferroptosis as manipulation of mitochondrial function may impact the outcome of these assays.^22^

#### 2.3.2 The role of lysosomes in ferroptosis

Lysosomes also play a role in ferroptosis induction. The lysosome is the major cellular ROS resource in erastin‐ or RSL3‐induced ferroptosis in HT1080 cells as detected by the fluorescence ROS sensors (Figure 1). Furthermore, the inhibitors of lysosome activity could prevent both the lysosomal ROS and a ferroptotic cell death‐associated ROS burst.^39^ Lysosome activity influences the intracellular iron provision by attenuating intracellular transport of transferrin or autophagic degradation of ferritin (Figure 1).^40^ The importance of the lysosome in ferroptosis has also been confirmed by another study from Gao et al They found that inhibition of lysosome cathepsin B, a cysteine proteinase, decreased the sensitivity of cells to erastin‐induced ferroptosis. STAT3 is involved in ferroptosis by regulating the expression of cathepsin B in human pancreatic ductal adenocarcinoma (PDAC) cell lines (Figure 1).^41^ A recent study recognized ferroptosis as an autophagic cell death which further reveals the important roles of lysosome in ferroptosis as lysosome is the major organelle for autophagic degradation of protein aggregates.^42^, ^43^, ^44^ Inhibition of autophagy by ATG13 and ATG3 knockdown greatly reduced cysteine deprivation‐induced ferroptosis.^39^ Autophagy may function to promote the ROS production and subsequent accumulation of lipid peroxides. Degradation of ferritin through NCOA4‐mediated ferritinophagy will release the iron for ferroptosis induction (Figure 1). NCOA4 knockdown decreases the ferritinophagy and leads to the unavailability of free iron, which abrogates the accumulation of ROS and decreases the ferroptosis induction.^45^ However, there is still lack of evidence to demonstrate ferroptosis as a direct consequence of autophagy.

#### 2.3.3 ER in ferroptosis

Endoplasmic reticulum stress is induced under various pathological conditions and is closely related to the cell death process. The ER stress responsive genes promote the apoptosis or autophagy process.^46^, ^47^ Erastin can also induce ER stress and up‐regulates ER stress responsive genes.^5^, ^48^, ^49^ The eif2α‐ATF4 branch is the major signalling pathway activated by ferroptotic reagents (Figure 1). CHAC 1 is the downstream of ATF4 and demonstrated to promote the degradation of GSH and the subsequent ferroptosis (Figure 1).^5^, ^50^ PUMA is another downstream of ATF4 and is also up‐regulated during the ER stress induced by the ferroptotic reagents, artemisinins (ART). However, PUMA activation will induce apoptotic cell death under the treatment of ferroptotic agents. What's more, knockout of PUMA did not reduce cell death by ART treatment alone. Only in combination treatment of ART and an apoptosis‐inducing ligand, TRAIL, did knockout of PUMA reduce cell death. So, the role of PUMA in ferroptosis is still elusive (Figure 1). Evidence showed that the ER stress induced by the ferroptotic reagents cannot be relieved through the inhibition of lipid peroxides by the Fer‐1 and Lip‐1.^49^ There is still little evidence to demonstrate the direct role of ER stress responsive genes in ferroptosis regulation.

### 2.4 Signalling pathways in ferroptosis regulation

In addition to those key ferroptotic initiation signals, multiple pathways are also involved in ferroptosis regulation. We summarize these ferroptotic signalling pathways associated with cancer progression and reveal its potential application in cancer therapy (Table 1). We also talked about several important signalling pathways in the following text.

**Table 1:** Regulators of ferroptosis in cancer cells

Effects	Regulators	Targets	Mechanisms	References
Ferroptosis promoters	p53	SLC7A11	System *x*_c_^−^ inhibition	54, 55, 56, 57
	HO‐1	Haem degradation	Cellular iron availability	62, 64
	antisense lncRNA as‐SLC7A11	SLC7A11	System *x*_c_^−^ inhibition	85
	G3BP1‐interacting lncRNA	p53 activation	System *x*_c_^−^ inhibition	86
	Hspb1	actin dynamics	Cellular iron availability	87
	FANCD2	GPX4; Iron metabolism genes	Cellular iron availability; GPX4 inhibition	65, 66
Ferroptosis inhibitors	miR‐137	glutamine transporter SLC1A5	Glutaminolysis	88
	Nrf2	Iron metabolism genes; SLC7A11; HO‐1	System *x*_c_^−^ inhibition; Cellular iron availability	51, 52
	p53	inhibition of DPP4 activity; activation of CDKN1A/p21	Lipid peroxidation; Cell circle arrest	54, 58
	HO‐1	/	/	59

#### 2.4.1 Nrf2

Nrf2 is a transcription factor that regulates iron metabolism genes in response to oxidative and electrophilic stress. Activation of Nrf2 promotes iron storage, reduces cellular iron uptake and limits ROS production. Thus, Nrf2 negatively regulates ferroptosis and promotes cancer progression. In HCC cell lines, p62 binds with Keap1 and disrupts the Keap1‐Nrf2 interaction upon exposure to ferroptosis‐inducing compounds. The disruption of the Keap1‐Nrf2 interaction stabilizes Nrf2 and promotes Nrf2 nuclear accumulation following treatment with ferroptosis‐inducing compounds, which decreases the sensitivity of cancer cells to ferroptosis induction.^51^ SLC7A11, a key component of system *x*_c_^−^, is also a target of Nrf2 and is up‐regulated when Nrf2 is activated.^52^ ARF is a tumour suppressor gene that activates p53 in tumour cells. However, ARF can directly inhibit the transcriptional role of Nrf2 and suppress its target genes, including SLC7A11, independent of p53. Loss of ARF activates Nrf2 and promotes cancer progression.^53^

#### 2.4.2 P53

P53 is a tumour suppressor gene that is activated under different stress stimuli. P53 is involved in ferroptosis as a transcriptional repressor of SLC7A11, impairing cysteine import and promoting ferroptosis initiation.^54^, ^55^, ^56^ P53 is also involved in other programmed cell death processes. However, the mechanism of p53 in ferroptosis induction is specific and different from other already known programmed cell death. An acetylation‐defective p53 mutant, p53^3KR^, has been created with 3 lysine residues replaced by arginine residues.^57^ This mutant is highly effective in repressing SLC711A expression but not that of other already known p53 target genes (cell cycle, apoptosis or senescence‐related genes). However, the acetylation‐defective form of p53, p53^4KR98^ is unable to inhibit SLC711A expression, while this mutated form can still repress the other p53 target genes.^54^ GLS2 is another p53 target gene involved in the regulation of ferroptosis and the promotion of p53‐dependent ferroptosis. MDM2 is an E3 ligase for the ubiquitination and degradation of p53. The *p53^+/+^Mdm2^−/−^* mouse embryos die at days E3.5‐E5.5 for the activation of p53. However, the *p53^3KR/3KR^ Mdm2^−/−^* embryos also showed obvious developmental abnormalities at days E11.5 without apoptosis induction, cell cycle arrest or cell senescence. The ferroptosis inhibitor can partially rescue these developmental defects in the *p53^3KR/3KR^ Mdm2^−/−^* embryos. These results indicated the potential role of ferroptosis in the embryonic development.^54^ However, there is also evidence showing that p53 could inhibit ferroptosis through inhibition of DPP4 activity or by the transcriptional activation of CDKN1A/p21, implying the dual roles of p53 in ferroptosis induction under different conditions.^58^

#### 2.4.3 Haeme oxygenase‐1

Haeme oxygenase‐1 can be regulated both by the transcriptional factor Nrf2 and the endoplasmic reticulum‐associated degradation pathway (ERAD).^59^, ^60^ Enhanced HO‐1 activity was shown to increase the cellular iron levels.^61^ The up‐regulation of HO‐1 can enhance haem degradation and change intracellular iron distribution. Both erastin and RSL3 induce the expression of HO‐1.^62^ Evidence from HO‐1 knockout mice or inhibition of HO‐1 by zinc protoporphyrin IX shows that HO‐1 promotes erastin‐induced ferroptosis.^63^ HO‐1 activation triggers ferroptosis through iron overloading and excessive ROS generation and lipid peroxidation.^64^ However, the role of HO‐1 in ferroptosis regulation is more complex. HO‐1 was also reported to function as a negative regulator in erastin‐ and sorafenib‐induced hepatocellular carcinoma ferroptosis as knockdown of HO‐1 enhanced cell growth inhibition by erastin and sorafenib. A similar result was also observed in renal proximal tubule cells. Immortalized renal proximal tubule cells obtained from *HO‐1^−/−^* mice administered with erastin and RSL3 had more pronounced cell death than those cells from wild‐type mice.^62^ These results suggest a dual role of HO‐1 in ferroptosis induction.

#### 2.4.4 FANCD2

Ferroptosis is involved in bone marrow injury caused by the traditional cancer therapy. FANCD2 is a nuclear protein involved in DNA damage repair, and its role in ferroptosis induction during the bone marrow injury was recently validated.^65^ FANCD2 was found to protect against ferroptosis in bone marrow stromal cells. Erastin treatment increased the protein levels of FANCD2, which protected against the DNA damage induced by erastin. FANCD2 can also influence the expression of a wide range of ferroptosis related genes, including the iron metabolism genes and GPX4. These findings highlight FANCD2 in ferroptosis inhibition, and the development of therapeutic strategies based on FANCD2 will benefit patients suffering from the side‐effects of cancer treatment.^66^

#### 2.4.5 BECN1

BECN1 is a key regulator of macroautophagy and functions during the early autophagy induction step for the formation of the autophagosome. Recent findings revealed a novel role of BECN1 in participation in the ferroptosis induction through system *x*_c_^−^ inhibition in cancer cells. BECN1 interacts with SLC7A11, the key component of system *x*_c_^−^, depending on the phosphorylation status by AMPK at S90/93/96 (Figure 1). The interaction between BECN1 and SLC7A11 inhibits the activity of system *x*_c_^−^, prevents the cysteine import and leads to the subsequent ferroptosis. In vivo tumour xenograft assays also demonstrate the anti‐tumour effect of BECN1 by inducing ferroptosis. Phosphorylation of BECN1 by AMPK at T388 promotes the BECN1‐PIK3C3 complex formation in autophagy.^67^ The different phosphorylation site of BECN1 by the AMPK will determine whether BECN1 will engage in BECN1‐SLC7A11 or BECN1‐PIK3C3 complexes to stimulate ferroptosis or autophagy, respectively. These findings suggest the dual roles of BECN1 in both autophagy induction and ferroptosis induction.^68^

### 2.5 Small molecule inducers of ferroptosis

Ferroptosis was originally defined during a chemical screen for cancer treatment. With increased research on ferroptosis, more ferroptosis‐inducing compounds have been identified. We summarize the existed compounds in ferroptosis induction in Table 2 and its applications in different cancer cells in Table 3.

**Table 2:** Ferroptosis‐inducing compounds

Reagents	Target	Mechanisms	References
Erastin and its analogs	System *X*_C_^−^; VDAC2/3	Cysteine deprivation;	1
RSL3	GPX4	GPX4 inactivation and GSH deletion	1, 8
Sulphasalazine	System *X*_C_^−^	cysteine deprivation	89
Sorafenib	System *X*_C_^−^	cysteine deprivation	5
ML162, DPI compounds	GPX4	GPX4 inactivation and GSH deletion	90
BSO, DPI2	GHS	GHS deletion	8
FIN56	CoQ10 and GPX4	CoQ10 deletion and GPX4 inactivation	91
FINO2	GPX4	GPX4 inactivation and lipid peroxides accumulation	92
Statins	HMG	CoQ10 deletion	93
Trigonelline, brusatol	Nrf2	Nrf2 inhibition	58
Siramesine, lapatinib	Ferroportin, Transferrin	increased cellular iron	94
BAY 87‐2243	Mitochondrial respiratory chain	Inhibition of mitochondrial respiratory chain (CI)	95
Cisplatin	GSH	Decreased GSH levels and GPXs inactivation	96
Artemisinins	Iron‐related genes	Increased cellular iron levels	71

**Table 3:** Cancer cells sensitive to ferroptosis

Cancer cells	Ferroptotic compounds	Type of evidence	References
Renal cancer cells	Sorafenib, erastin, RSL3, BSO	Cell culture, mice model, tissues from patients	8
Human hepatocellular carcinoma	Erastin, sorafenib, DPI compounds, trigonelline, brusatol	Cell culture, tumour xenograft model	1, 97
Breast cancer cells	Erastin, siramesine, lapatinib	Cell culture, tumour xenograft model	69
Pancreatic cancer cells	Erastin, sorafenib, artesunate	Cell culture	70, 71
Human non‐small cell lung cancer	Sorafenib, erastin, RSL3, M162	Cell culture	96
Diffuse large B‐cell lymphomas	Sulphasalazine, erastin, RSL3	Cell culture	8
Glioma cells	Erastin, sulphasalazine, RSL3,	Cell culture	72, 75
Ovarian cancers	Erastin	Cell culture, tumour xenograft model, cancer cells from patients	76, 77
Colorectal cancers	Cisplatin, erastin,	Cell culture, tumour xenograft model	49, 58, 96
Acute myeloid leukaemia	Erastin,	Cell culture	98
Acute lymphoblastic leukaemia	RSL3	Cell culture	99
Rhabdomyosarcoma cells	Erastin, RSL3	Cell culture	15
Human cervical cancer cells	Erastin	Cell culture	87
Prostate cancer cells	Erastin	Cell culture	80
Osteosarcoma cells	Erastin	Cell culture	73
Head and neck cancer	Erastin, sulphasalazine,	Cell culture, tumour xenograft model	74, 100
Melanoma	BAY 87‐2243, erastin, RSL3	Cell culture, tumour xenograft model	20, 80
Glioblastoma	Erastin,	Cell culture	74
Fibrosarcoma cell	Sulphasalazine, erastin, BSO, RSL3, DPI2, FIN56, FINO2, statins	Cell culture, mouse xenograft model	40, 64, 96

### 2.6 Ferroptosis regulation in different cancers

Although the precise mechanism that determines the ferroptosis sensitivity in cancer cells is largely unknown, cancer cells from different tissues show different degrees of ferroptosis sensitivity. We discussed the sensitivity of ferroptosis to the ferroptotic reagents and the general initiation mechanism of ferroptosis above, and next, we talk about the specific mechanisms of ferroptosis induction in several special types of cancer cells.

#### 2.6.1 Ferroptosis in breast cancer cells

Breast cancer cells seem less sensitive to the ferroptotic reagents (Erastin, RSL3, ML210 and ML162). However, studies have shown that both the lysosome disrupting agent (siramesine) and the tyrosine kinase inhibitor (lapatinib) could induce ferroptosis in breast cancer cells. Mechanistic studies have demonstrated that siramesine and lapatinib increased cellular iron levels by up‐regulating the expression of transferrin and down‐regulating the expression of ferroportin‐1. The cell death could be rescued by the ferroptosis inhibitors Fer‐1.^69^ However, ferroportin‐1 overexpression failed to affect erastin‐induced cell death. Moreover, siramesine induced the release of cathepsin B from the lysosome, which is a cysteinase that consumes the cellular cysteine. Lapatinib is an inhibitor of EGFR and HER2; however, silencing of EGFR and HER2 could not rescue lapatinib‐induced ferroptosis. This suggests that there were different targets of lapatinib during ferroptosis induction.

#### 2.6.2 Ferroptosis in pancreatic cancer cells

Pancreatic ductal adenocarcinoma (PDAC) cells transformed by KRAS are highly resistant to apoptosis and are almost an incurable cancer at present. The development of therapeutic strategies for this cancer is a challenge in the clinical practice. Pancreatic cancer cells seem also insensitive to common ferroptosis reagents. However, studies have shown that artesunate (ART) induces ferroptosis in PDAC in a ROS and iron‐dependent manner. Ferroptosis inhibitor, Fer‐1, but not the apoptosis or necrosis inhibitors, can block ART‐induced lipid peroxidation and cell death in PDAC. ART may promote ferroptosis induction through modulating the expression of iron‐related gene, which contribute to ferroptotic cells death. These findings provide a promising way for PDAC treatment.^70^, ^71^

#### 2.6.3 Ferroptosis in lymphomas and renal cancers

In a study performed by Wan Seok Yang,^8^ they found that erastin had similar lethality in both the RAS‐mutated cancer cell lines and the RAS wild‐type cells. However, certain types of cancer cells showed great sensitivity to ferroptosis induction. Diffuse large B‐cell lymphomas are the most sensitive to erastin‐induced ferroptosis, while diffuse large B‐cell lymphomas induction is insensitive to other lethal compounds.^8^ This increased sensitivity might be attributable to a deficiency of the sulphur‐transfer pathways in some types of leukaemia and lymphoma. The ferroptosis sensitivity also depends on the tissue type. Renal cell carcinomas showed increased sensitivity to erastin‐induced ferroptosis in diverse tissues. The deep understanding of these genetic features in cancer cells most sensitive to ferroptosis will help to improve the therapy strategy based on ferroptosis induction.^8^

#### 2.6.4 Ferroptosis in brain tumours

The nervous system contains the highest content of PUFAs in our body, which are the main substrates for the production of peroxides. Brain tumours were more sensitive to ferroptosis induction. Both erastin and sorafenib can induce potent cell death in malignant brain tumours. However, brain tissues also develop a protection system against cell death. Increased Nrf2 activation was also observed in brain tumours. The Nrf2‐Keap1 pathway protects cancer cells from ferroptosis induction as mentioned above. In glioma cells, Nrf2 overexpression or Keap1 knockdown promotes the oncogenic transformation. Nrf2 up‐regulation in patients with brain tumours also showed reduced survival rate. Glioblastoma (GBM) is a very aggressive brain tumour with poor prognosis. Even after surgery and radiochemotherapy, the cancer invariably recurs and leads to patient death. Cancer stem cells (CSCs) have been suggested to play a role in refractory/relapsing cancers. Temozolomide (TMZ) was used for GBM treatment. TMZ selectively induced GSCs to undergo ferroptosis but not apoptosis or necrosis in TMZ‐induced cell death during treatment. That result also showed the sensitivity of ferroptosis induction in brain tumours.^72^, ^73^, ^74^, ^75^

#### 2.6.5 Ferroptosis in ovarian cancers

Ovarian cancer is the most dangerous gynaecological malignancy in women. Most patients develop recurrent, chemo‐resistant and ultimately terminal diseases. CSCs have been linked to the development of recurrence and resistance to therapy in ovarian cancer. Recent studies have shown that CSCs in ovarian cancer rely on iron for proliferation and evasion. However, this phenomenon provides an opportunity for treating cancer cells with agents that induce ferroptosis. It has been reported that CSCs were significantly more susceptible to erastin treatment than non‐cancer stem cells.^76^, ^77^

### 2.7 The potential application of ferroptosis in overcoming cancer cells' drug resistance

Cancer cells' resistance to chemotherapy is a major problem in cancer treatment. The ineffective induction of cell death is one of the features shared by most chemotherapy drugs. As ferroptosis is a totally different cell death process from apoptosis, ferroptotic reagents may represent a promising strategy in overcoming the inefficiency of apoptosis‐inducing chemotherapy drugs in cell death induction. Efforts have been made to explore the application of inducing ferroptosis in overcoming the cancer cells' drug resistance.

#### 2.7.1 Ferroptosis promotes the cell death of drug‐tolerant cancer cells with mesenchymal state

**Figure 3:**
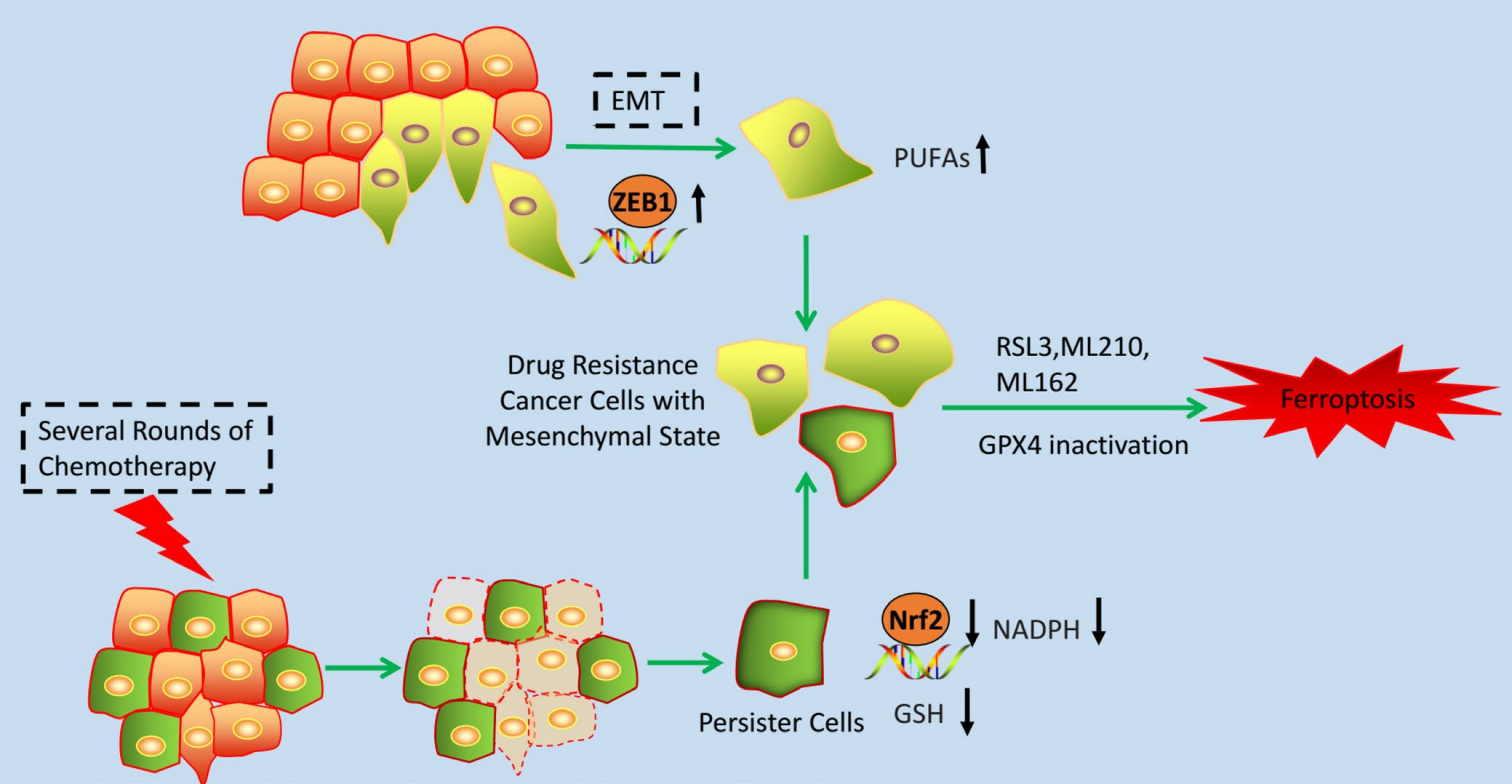
Potential application of ferroptosis in overcoming cancer cells' drug resistance. EMT process promotes the mesenchymal state of cancer cells through the activation of ZEB1. The surviving cells (persister cells) after several rounds of chemotherapy obtained the mesenchymal features. Nrf2 target genes are down‐regulated, and the levels of NADPH and GSH are decreased in these cells with mesenchymal state. GPX4 inactivation is lethal to cancer cells with mesenchymal state

Transition of epithelial cancer cells into a mesenchymal state via epithelial to mesenchymal transition (EMT) is a process that brings multiple mechanisms of resistance to cell death including the inactivation of apoptotic programmes across a large range of cancer cells.^78^, ^79^ Insight into vulnerabilities of these cancer cells with mesenchymal state is a promising way to improve the therapeutic strategy. Studies showed that cancer cells with mesenchymal state harboured a higher activity of enzymes that promote the synthesis, storage and use of long‐chain PUFAs, which are the sources of reactive lipid peroxides, making these cancer cells highly dependent on GPX4 for survival (Figure 3).^80^ This vulnerability makes it possible to induce ferroptosis in these cancer cells through inhibition of GPX4. In fact, the ferroptotic reagents are demonstrated to strongly correlate with the selective cell death of epithelial‐derived cancer cells with high‐mesenchymal state (Figure 3).^80^ However, the artificially induced mesenchymal state cancer cells by overexpression of SNAIL1 or TWIST does not show this sensitivity to ferroptotic reagents. Mechanism study shows that ZEB1, which functions both in the EMT and lipogenic process, links the mesenchymal state with the lipidperoxide vulnerability.^80^, ^81^

These results can be verified in different types of cancer cells. Evidence has shown that HCC4006 non‐small cell lung cancer cells with a high‐mesenchymal state are resistant to gefitinib. However, these same cells were preferentially sensitive to GPX4 inhibition compared with parental cells. Cancer cells with mesenchymal origin also showed great sensitivity to ferroptotic compounds.^80^

#### 2.7.2 Ferroptosis promotes the cell death of the drug‐tolerant persister cancer cells

The potential application of ferroptosis in overcoming cancer cells' drug resistance can also be reflected from its role of inducing cell death of persister cells. Persister cells are the surviving cancer cells upon treatment with several rounds of chemotherapy drug, which is another therapy‐resistant cell state presented across a wide range of tumour types (Figure 3).^82^, ^83^ Targeting the persister cancer cells is also an important strategy for overcoming cancer cells' drug resistance. The stemness markers and mesenchymal markers are up‐regulated in the persister cells, showing the mesenchymal state of these cancer cells.^84^ The exploration of the vulnerability of these cancer cells shows that the Nrf2 target genes were down‐regulated. As we mentioned above, Nrf2 is a major suppressor of ferroptosis. Further study showed that persister cells have markedly decreased levels of both glutathione and NADPH and have a specific sensitivity to lipid peroxidation rather than general sensitivity to oxidative stress. Evidence shows that GPX4 inhibitors are specifically lethal in the persister cells through ferroptotic cell death (Figure 3).^80^, ^84^ Based on these results, inducing ferroptosis may be a promising way to overcome these cells' drug resistance.

## 3 DISCUSSIONS

Programmed cell death is a hot topic both in biological research and medicine. Targeting cell death process is the common way in cancer treatment. As a new coined programmed cell death process, ferroptosis is characterized with unique features and shows great potentials in the cancer therapy. Although excellent progress has been made in the recent years, there are still open questions remained to be answered. Firstly, what is the developmental significance of ferroptosis? There are some clues indicating the role of ferroptosis in development. However, it is still largely elusive whether these cell deaths are ferroptotic process. We lack the markers for labelling ferroptotic cell death in vivo. Secondly, it is one of the criteria for the definition of programmed cell death that the execution of cell death is modulated by certain gene product. Although it is widely accepted that lipid peroxides are the causative factors for the ferroptotic cell death, the exact executor of ferroptosis is unknown. Next, iron dependency is the fundamental property of ferroptosis, but the exact role of iron in this process is still elusive. The Fenton reaction may explain the role of iron in promoting ROS production. However, this reaction is not specific to the iron. Other metal irons also contain this feature but are not responsible for the ferroptotic induction. Ferroptosis‐inducing compounds are exclusively effective to certain cancer cells but not others. Efforts are still needed to classify the types of cancers which are sensitive to ferroptosis. This is important for the application of ferroptosis to cancer therapy. Moreover, the relationship between ferroptosis and other cell death process needs to be classified clearly under different pathological conditions because it is important to combine different methods to the diseases treatment.

## CONFLICT OF INTEREST

The authors confirm that there are no conflicts of interest.

## AUTHOR CONTRIBUTION

TX and JW provided direction and guidance throughout the preparation of this manuscript. TX, WD, XJ, XA and YL collected and prepared the related literature. TX and JW drafted the manuscript. WY and JW reviewed and made significant revisions to the manuscript. All authors have read and approved the final manuscript.
